# Structural effects and potential changes in growth factor signalling in penis-projecting autonomic neurons after axotomy

**DOI:** 10.1186/1471-2202-7-41

**Published:** 2006-05-23

**Authors:** Catalina A Palma, Janet R Keast

**Affiliations:** 1 Prince of Wales Medical Research Institute, University of New South Wales, Sydney NSW, Australia; 2 Pain Management Research Institute, Kolling Institute of Medical Research, University of Sydney at Royal North Shore Hospital, St Leonards NSW 2065, Australia

## Abstract

**Background:**

The responses of adult parasympathetic ganglion neurons to injury and the neurotrophic mechanisms underlying their axonal regeneration are poorly understood. This is especially relevant to penis-projecting parasympathetic neurons, which are vulnerable to injury during pelvic surgery such as prostatectomy. We investigated the changes in pelvic ganglia of adult male rats in the first week after unilateral cavernous (penile) nerve axotomy (cut or crush lesions). In some experiments FluoroGold was injected into the penis seven days prior to injury to allow later identification of penis-projecting neurons. Neurturin and glial cell line-derived neurotrophic factor (GDNF) are neurotrophic factors for penile parasympathetic neurons, so we also examined expression of relevant receptors, GFRα1 and GFRα2, in injured pelvic ganglion neurons.

**Results:**

Axotomy caused prolific growth of axon collaterals (sprouting) in pelvic ganglia ipsilateral to the injury. These collaterals were most prevalent in the region near the exit of the penile nerve. This region contained the majority of FluoroGold-labelled neurons. Many sprouting fibres formed close associations with sympathetic and parasympathetic pelvic neurons, including many FluoroGold neurons. However immunoreactivity for synaptic proteins could not be demonstrated in these collaterals. Preganglionic terminals showed a marked loss of synaptic proteins, suggesting a retrograde effect of the injury beyond the injured neurons. GFRα2 immunofluorescence intensity was decreased in the cytoplasm of parasympathetic neurons, but GFRα1 immunofluorescence was unaffected in these neurons.

**Conclusion:**

These studies show that there are profound changes within the pelvic ganglion after penile nerve injury. Sprouting of injured postganglionic axons occurs concurrently with structural or chemical changes in preganglionic terminals. New growth of postganglionic axon collaterals within the ganglion raises the possibility of the formation of aberrant synaptic connections between injured and un-injured ganglion neurons. Together these changes demonstrate a broader effect on the pelvic autonomic circuitry than simply loss of neuroeffector connections. These structural changes are accompanied by potential changes in neurotrophic factor signalling due to altered expression of receptors for members of the GDNF family. Together our results advance understanding of the responses of pelvic autonomic nerve circuits to injury and may assist in designing strategies for promoting regeneration.

## Background

In contrast to neurons of the central nervous system, many adult peripheral neurons not only survive axotomy but also undergo axonal regeneration. Within just a few hours, injured axons grow collaterals ("sprouts") and their growth cones respond to guidance cues in order to find an environment supportive of regeneration [1-3]. As well as being regarded as the beginning of the regenerative response, many sprouts form close associations with neighbouring ganglion neurons. These have been best described for sympathetic ganglion and primary sensory (dorsal root ganglion) neurons [4-6]. Neurotrophic factors, released by target tissues and by glial cells in the vicinity of the injured neuron, are critical for this growth [3,7]. In sympathetic ganglia the neurotrophins, especially nerve growth factor, play this role after injury. Neurotrophin-dependent regeneration is also associated with changes in expression of neurotrophin receptors, possibly caused by altered availability of ligand, and setting up a feedback loop in the regenerative process [8-10].

The regenerative abilities of injured parasympathetic ganglion neurons are very poorly understood. This is partly because of technical difficulties in selectively injuring their axons, given that most parasympathetic neurons lie in small, dispersed ganglia that are closely associated with or even within their target tissues [11]. Therefore, damage to many organs injures not only the axons but also the somata of parasympathetic ganglion neurons. Another major factor has been the delay in identifying the neurotrophic factors for parasympathetic neurons. Neurturin (NTN) belongs to a recently identified family of neurotrophic factors, which include glial cell line-derived neurotrophic factor (GDNF), NTN, artemin and persephin [12-14]. NTN and GDNF are important during the development (survival) and/or maintenance of many cranial parasympathetic neurons. Their actions vary with the stage of development and between different cranial ganglia [15-18]. The GDNF family ligands (GFLs) act via a two-component receptor complex, a common signalling component, the Ret receptor tyrosine kinase, and a glycosylphosphatidylinositol-anchored GFRα protein, which binds ligand with high affinity and determines specificity [14]. GDNF preferentially binds to GFRα1 and NTN to GFRα2, although *in vitro *studies have suggested that each ligand may be able signal through alternative GFR mechanisms at higher concentrations [13,19].

NTN is a neurotrophic factor for many pelvic cholinergic ganglion neurons, which are the sole source of parasympathetic innervation of the urogenital organs and a major source of extrinsic autonomic nerves supplying the lower bowel [20]. Most or all of the pelvic organs express NTN [21-23] and most adult cholinergic pelvic neurons express GFRα2 [24]. Mice deficient in GFRα2 exhibit loss of cholinergic nerve terminals in some regions of the penis [22]. These mice, and *neurturin *knockout mice, also show a substantial decrease in cholinergic innervation of reproductive organ epithelia [24]. Both deficits may indicate a failure of survival, arborisation or maintenance of these projections. Recently, NTN has been shown to have multiple neurotrophic actions on adult rat pelvic parasympathetic ganglion neurons *in vitro *[25]. GDNF is also relevant to maintenance of penile innervation; many penile neurons express GFRα1 and retrogradely transport GDNF from the penis [22].

Pelvic autonomic neurons, especially parasympathetic neurons, are inadvertently axotomised during pelvic surgical procedures such as prostatectomy, hysterectomy, and bowel resections for cancer removal [26-28]. This commonly leads to post-operative urogenital problems such as erectile dysfunction and urinary incontinence. There is high demand for new regenerative strategies for these injured neurons. As an initial step in understanding the injury and regenerative processes in these neurons, we have investigated whether pelvic parasympathetic neurons undergo similar changes after axotomy as seen in adult sympathetic and dorsal root ganglia, by assessing structural changes in adult rat pelvic ganglia shortly after penile (cavernous) nerve transection or crush. To provide further insight into the role of growth factors in the regenerative response we investigated the effects of axotomy on the neuronal expression of the NTN receptor, GFRα2, and the GDNF receptor, GFRα1. We chose to use immunohistochemical analysis for this component of the study as it allows changes in neuronal expression to be distinguished from those in non-neuronal cells, such as glia, and also allows expression to be monitored in different chemical classes of neurons or neurons retrogradely labelled from the penis. Our results show profound structural changes in the pre- and postganglionic nerve pathways innervating the penis following penile nerve injury. We also identify an effect of axotomy on GFRα2 expression by injured neurons, suggesting an effect of injury on growth factor signalling.

## Results

### Axotomy of penile neurons causes structural changes in pelvic ganglia

We used two immunohistochemical markers to monitor changes in pelvic ganglion neurons after nerve injury. VIP (vasoactive intestinal peptide), is a marker for many parasympathetic ganglion neurons [29] and is expressed by virtually all pelvic parasympathetic neurons innervating the penis [30-33]. TH (tyrosine hydroxylase) is a marker of noradrenergic sympathetic pelvic ganglion neurons [29] but is not expressed by any pelvic ganglion penis-projecting neurons [32].

**Figure 1:**
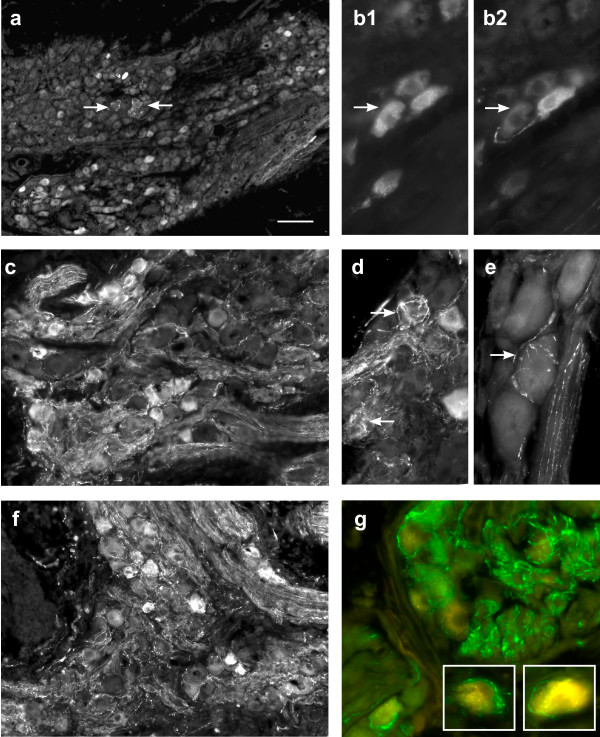
**Structural changes in rat pelvic ganglia after penile nerve transection**. All micrographs show immunostaining for VIP. In a, c and f the region of the ganglion closest to the penile nerve is at the bottom left of the micrograph. Panels b-g are from injured ganglia; all show ganglia ipsilateral to penile nerve transection except for panel f (penile nerve crush). a. Uninjured (contralateral) ganglion. VIP neurons are found throughout the ganglion but are clustered in the region near the penile nerve exit; VIP baskets are sparse. b. FluoroGold (FG) neurons retrogradely labelled from the penis (b1), immunoreactive for VIP (b2); the axon from one FG neuron appears to encircle an adjacent neuron (arrows). c. Numerous varicose VIP axons growwithin the ganglion ipsilateral to the injury, especially in theregion closest to the penile nerve (bottom left). d, e. Examples ofVIP baskets encircling pelvic ganglion neurons in the ganglion ipsilateral to penile nerve transection. f. Similar growth of VIP axon collaterals occurs after penile nerve crush. g. FluoroGold-labelled neurons surrounded by varicose VIP collaterals. Calibration bar represents: 100 μm (a), 50 μm (c, f), 20 μm (b, d, e, g) or 15 μm (g inset).

In the present study, VIP neurons were distributed throughout the pelvic ganglion but were particularly prevalent near the dorsal region where the penile nerve exits, as described previously [31,34]. In pelvic ganglia from unlesioned animals, VIP baskets were sparse and most are known to comprise projections from viscerofugal neurons of the lower bowel [35]. Eight days after unilateral penile nerve axotomy (transection), VIP baskets were still sparse in the pelvic ganglia contralateral to the injury (Fig. 1a). In contrast, in the ipsilateral (i.e. injured) ganglion many VIP fibres (putative axon collaterals) were present and were concentrated in the dorsal region near the exit of the penile nerve (Fig. 1c). Many of these fibres appeared to form discrete "baskets" surrounding a subgroup of pelvic ganglion neurons (Fig. 1d, e). Studies where the retrograde tracer, FluoroGold, was injected into the penis seven days prior to nerve injury showed that many penis-projecting neurons were targeted by these injury-induced fibres (Fig. 1g). Ipsilateral to the injury, 38.5 ± 2.1% of the FG-labelled neurons were targeted by these new fibres (n = 5 animals), whereas in the contralateral ganglion only 5.3 ± 1.0% of FG-labelled somata (n = 5 animals) were surrounded by VIP baskets. VIP fibre growth appeared very similar in ganglia following a crush injury to the penile nerve (Fig. 1f), but was not quantified. In rats more than 90% of retrogradely-labelled penile neurons in the pelvic ganglion express VIP [31,32] and we also observed that after either type of penile nerve injury most FG neurons continued to express VIP (Fig. 1b).

**Figure 2:**
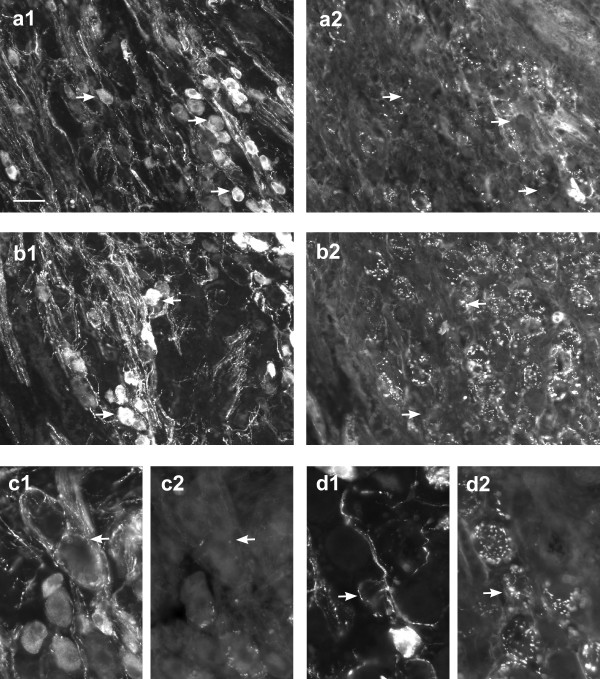
**Expression of the synaptic protein, synaptophysin, in injured pelvic ganglia**. All pairs of micrographs showdouble-staining immunofluorescence for VIP (left) and synaptophysin(right). Panels a and b are from ganglia where the penile nerve wascut, whereas panels c and d are from penile nerve crush experiments. a, b. Synaptophysin-positive terminals are significantly reduced nearsome clusters of VIP neurons (many of which would be expected to beinjured) and in regions where there are many VIP fibres. Matchingpairs of arrows show examples of VIP somata. c, d. Little or nosynaptophysin expression is present in VIP fibres. Examples of VIP-positive, synaptophysin-negative fibres are indicated by arrows. Calibration bar represents: 40 μm (a, b) or 20 μm (c, d).

Preganglionic terminals in pelvic ganglia can be visualised by immunostaining for synaptic proteins [36,37]. Following either penile nerve cut (Fig. 2a, b) or crush (Fig. 2c, d) injuries, there was a marked reduction in synaptophysin- or synapsin-immunoreactive varicosities surrounding somata in the region near the exit of the penile nerves, i.e. where penis-projecting neurons are prevalent (Fig. 2a, b). This suggests that postganglionic axotomy caused a loss of preganglionic terminals (or decrease in synaptic protein expression) in newly growing axon collaterals. Double-staining immunofluorescence showed that the residual fibres expressing synaptic proteins did not co-express VIP (Fig. 2c, d), indicating that the new axon collaterals initiated by the injury process are unlikely to make functional synapses by this time.

### Axotomy causes a decrease in GFRα2 but not GFRα1 expression in penis-projecting pelvic neurons

**Figure 3:**
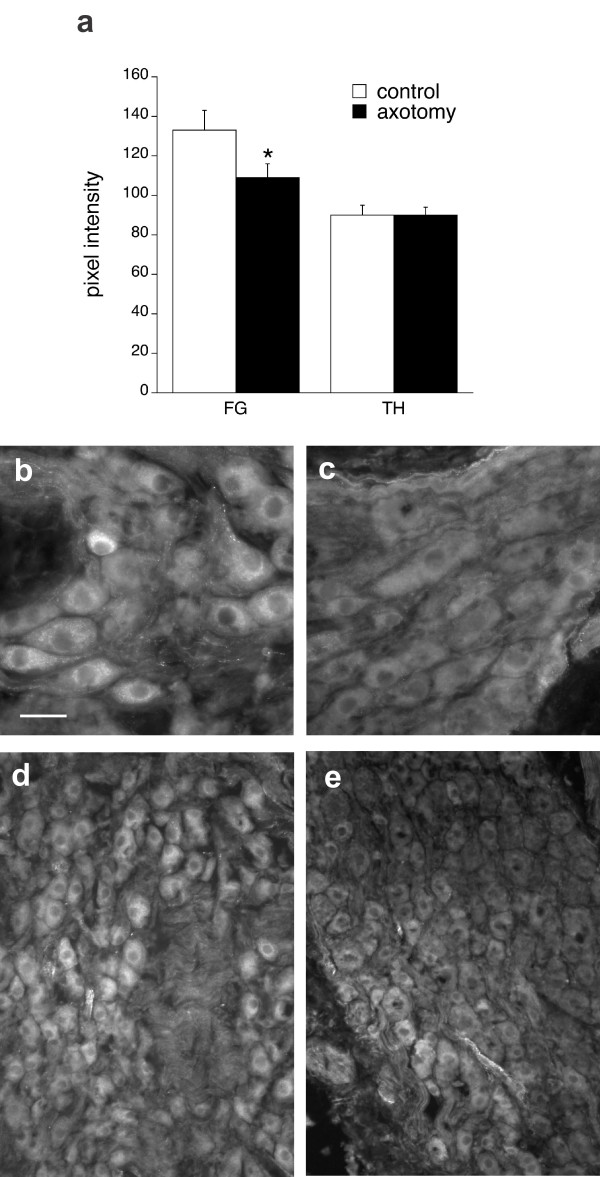
**Effects of axotomy on intensity of GFRα2immunofluorescence in rat pelvic ganglia**. a. Immunofluorescence intensity measurements showed that GFRα2 immunoreactivity decreased in injured FluoroGold (FG) neurons (*P *= 0.0068) but not in FG neurons contralateral to the injury ("control") or in noradrenergic neurons (TH, tyrosine hydroxylase). Data were obtained from 5 rats that underwent unilateral penile nerve transection, with matched data from left (injured) and right (control) ganglia from the same animal. b, d. Pelvic ganglion contralateral to penile nerve transection. Manyneurons showed GFRα2-immunostaining. c., e. Pelvic ganglionipsilateral to penile nerve transection. GFRα2 immunofluorescence intensity is very low in the majority of neurons, although some small groups of immunostained neurons are still present. Calibration bar represents: 20 μm (b, c) or 40 μm (d, e).

GFRα2-immunoreactivity was present in many neurons in the rat pelvic ganglion, as described previously [24]. This was evident as fine granular staining in the cytoplasm and occasionally staining of the plasma membrane. As described previously, most GFRα2-positive neurons were immunopositive for VIP but rarely expressed TH, and conversely the majority of VIP pelvic neurons expressed GFRα2 [24]. Quantitation of GFRα2 immunofluorescence intensity in FG neurons (known to express VIP) and in TH neurons from pelvic ganglia contralateral to the injury demonstrated this difference in expression level (Fig. 3a, "control").

After axotomy, GFRα2 staining intensity decreased in many pelvic ganglion neurons ipsilateral to the injury (Fig. 3b–e). Analysis of GFRα2 immunofluorescence intensity in FG neurons showed that axotomy caused a decrease in GFRα2 expression in injured neurons (Fig. 3a, "injured"). There was no change in GFRα2 expression within TH (noradrenergic) neurons of the ipsilateral ganglion, none of which were labelled with FG. Penile nerve transection would not be predicted to damage these neurons.

**Figure 4:**
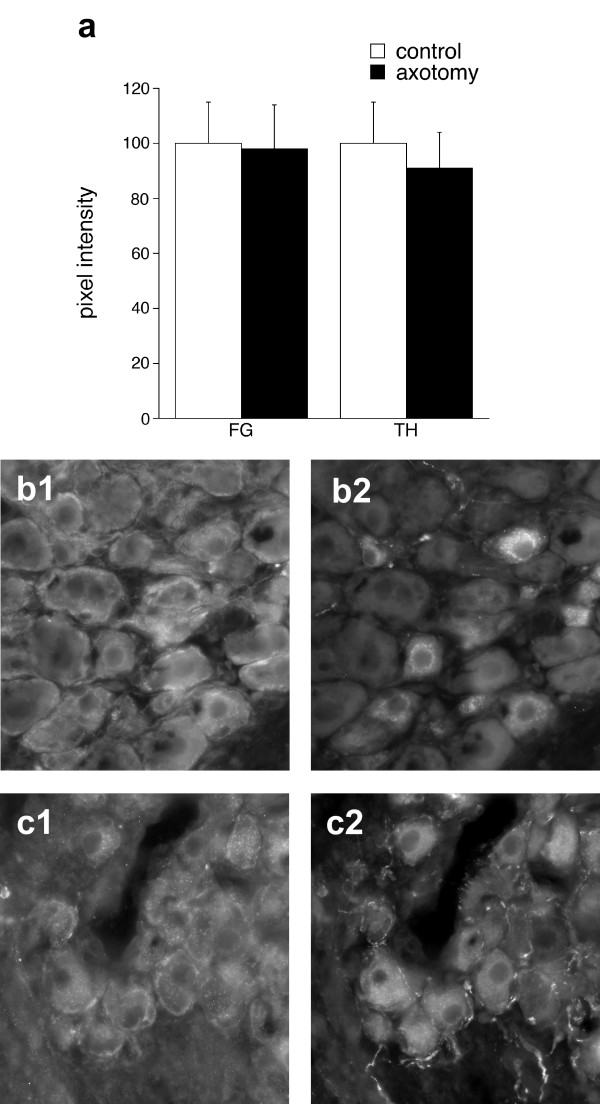
**Effects of axotomy on intensity of GFRα1immunofluorescence in rat pelvic ganglia**. a. Immunofluorescence intensity measurements showed that GFRα1 immunoreactivity was not affected in injured FluoroGold (FG) but decreased slightly in noradrenergic neurons (TH; *P *= 0.046). Data were obtained from 5 rats that underwent unilateral penile nerve transection, with matched data from left (injured) and right (control) ganglia from the sameanimal. Panels b and c show matched pairs of images from sections double-stained for GFRα1 (left) and VIP (right). b, contralateral (control) ganglion. c, ipsilateral (injured) ganglion. Calibration bar represents 20 μm.

GFRα1 was more broadly expressed in pelvic ganglion neurons and expressed at similar levels in both VIP and TH neurons. Quantitation of GFRα1 immunofluorescence intensity in FG neurons (known to express VIP) and in TH neurons from pelvic ganglia contralateral to the injury demonstrated this similarity in expression level (Fig. 4a, "control"). Most neurons of each type showed low to moderate levels of GFRα1-immunoreactivity. GFRα1 immunostaining was present within the cytoplasm of neuronal somata, occasionally with some staining of the neuronal plasma membrane, glia and some fibres that may be axons or glial processes. Axotomy had no effect on GFRα1 expression in FG neurons (Fig. 4a, c), but GFRα1 fluorescence intensity decreased slightly in TH neurons, even though these neurons would not be injured by penile nerve transection (Fig. 4a). Figure 4b also shows that there is no obvious difference between VIP- positive and -negative neurons in GFRα1 fluorescence intensity.

## Discussion

We have examined the effects of axotomy on pelvic parasympathetic ganglion neurons and possible changes in growth factor receptor expression initiated by the injury. The penile nerve was chosen as it is a well-characterised projection from the pelvic ganglion, and is both easily identifiable and readily accessible for experimental intervention. It is also relevant to clinical problems associated with pelvic surgery (e.g. prostatectomy) where penile axons are injured and the demand for regenerative therapies is high. Our results show that axotomy causes sprouting within the pelvic ganglion, most likely from the axons of injured parasympathetic neurons. Moreover, axotomy may trigger structural or chemical changes in preganglionic terminals innervating the damaged neurons. Our results showed that following axotomy there is a decrease in GFRα2 but not GFRα1 expression in pelvic parasympathetic neurons, which may indicate a change in GFL signalling mechanisms at an early phase of the regenerative state.

The pelvic ganglion is unusual, not only because it contains intermingled populations of sympathetic and parasympathetic neurons, but also because many of its parasympathetic neurons lie distant from their target organs [20]. Moreover, a minority of cholinergic pelvic neurons receive synaptic inputs from the lumbar cord, so strictly speaking should be referred to as sympathetic [29]. This is also the case for a minority of penis-projecting pelvic cholinergic neurons [38], however in the present study we did not determine the source of spinal inputs for FG-labelled neurons. Because most of these are likely to be parasympathetic (i.e. sacral inputs) and in recognition of their similar chemical phenotype and likely growth factor dependency, for simplicity we have referred to them collectively as penile parasympathetic neurons.

In the present study we injured only one population of pelvic parasympathetic neurons but assessed the effects of injury on the broader population of parasympathetic neurons and on the neighbouring uninjured noradrenergic sympathetic populations. Most of our results concur with structural and chemical changes being restricted to the injured parasympathetic population, although a small decrease in GFRα1 expression in sympathetic neurons after penile nerve transection suggests that some changes may occur more broadly in the ganglion. One possible mechanism for this is if axotomy alters glial secretions or glial coupling within the ganglion, allowing signalling between pelvic ganglion neurons. This has been described in sensory ganglia [39].

Penile nerve injury caused sprouting of VIP neurons in the region of the ganglion closest to the injury site, resembling the sprouting seen in sympathetic ganglia after axotomy, and regarded as an early phase of the regenerative response [1,2]. The vast majority of rat pelvic ganglion neurons are adendritic or have only a few short fine dendrites [40]. Therefore potential cellular targets of growing fibres are relatively easy to identify. Following penile nerve lesion, VIP fibres formed basket-like structures around pelvic ganglion neurons, many of which were injured (i.e. FG-labelled). It is not known if these fibres are capable of forming functional synapses or are likely to be permanent, although eight days after injury did not yet express immunohistochemically detectable levels of synapsin or synaptophysin. Aberrant connections are made within other injured ganglia and are thought to underlie prolonged problems with some visceral reflexes [5,6,36]. In the pelvic ganglion these changes could lead to profoundly altered connectivity and functionality in the penile erection reflex circuitry following axotomy. Therefore, even if regeneration to the target occurs, other problems with reflexes may continue.

Our immunohistochemical studies raise the possibility of axotomy triggering dramatic changes within preganglionic axons that synapse on penis-projecting neurons. The loss of synaptic protein immunoreactivity within these terminals may represent a chemical or structural change, or both. Ultrastructural or electrophysiological methods are necessary to determine the functional nature of the deficit. There are various precedents for such a retrograde effect of neuronal injury. Previous studies of sympathetic ganglia show retraction of dendrites and of preganglionic inputs after postganglionic axotomy, both of which reverse if postganglionic regeneration occurs [41,42]. There are also profound and at least partially reversible changes in synaptic transmission, nicotinic receptor expression and postganglionic membrane properties after axotomy [43-46]. It is important to determine the longer-term impact of penile nerve injury on lumbosacral spinal neurons. The reversibility of this change with penile nerve regeneration or compensatory growth from ipsilateral pathways should also be determined in order to understand the possible extent of recovery or manipulation required to facilitate recovery in this system.

A number of groups have shown that penile erection can return after injury, although after bilateral transection the success of reinnervation is low [47-49]. Regeneration of the erectile response is much more successful after unilateral penile nerve lesion, indicating that it is largely due to compensatory sprouting of uninjured penile-projecting fibres. Both types of growth, regeneration and compensatory, may be driven by and depend on neurotrophic factors, the most likely candidates being NTN and GDNF. NTN is synthesised by many urogenital tissues, including the penis [22,50,51] and radioactively labelled NTN is transported retrogradely along penile axons [22]. Indeed, axotomy of penis-projecting neurons causes a decrease in soma size [25], consistent with reduced access to neurotrophic factors. However, NTN may also be secreted by glial cells within the ganglion or glia associated with the injured axon [52-54]. These would be the most likely sources of trophic factor after injuries where axons are disconnected from their target organs. Another interesting possibility has been raised by a recent report of NTN expression by immune cells [55].

GDNF [56] and neurotrophin-3 [57] are additional possible neurotrophic factors for penile parasympathetic neurons. In the present study all of the VIP-immunoreactive and FG-labelled neurons expressed GFRα1. GDNF can also prevent some of the axotomy-induced changes, including sprouting, in sensory neurons [58], and has additive effects with NTN to rescue retinal ganglion neurons from axotomy-induced death [59]. It is possible that a combination of these growth factors acts in concert to promote regenerative responses in the penis-projecting neurons.

The present study showed that penile nerve axotomy consistently caused a decrease in GFRα2 expression in injured neurons but had no effect on GFRα1, however our experiments do not exclude an effect on uninjured parasympathetic neurons. Previous experiments on dorsal root ganglion neurons showed that after sciatic nerve injury there was a decrease in GFRα2 but an increase in GFRα1 expression, and that GDNF was able to prevent the change in GFRα2 [60,61]. The greater effect of injury on GFRα1 shown in these earlier studies may indicate a fundamental difference between sensory and parasympathetic ganglia in their injury responses or in their GFL availability within injured ganglia. It is also possible that in our experiments subtle changes occurred in GFRα1 expression that were not detected with our technique. Our observation of a decrease in GFRα2 expression after axotomy does not necessarily preclude a role of NTN in axonal regeneration. For example, it is possible that NTN is involved in the initial extension of axons or collatorals following injury but then further axonal extension is NTN-independent. Alternatively, even after down-regulation of GFRα2 there may still be sufficient receptor expression to mediate physiological maintenance and growth responses. Ideally the expression of NTN and GFRα2 should be monitored or manipulated at various time points following injury. In addition, our studies relied on immunohistochemistry to assess changes in GFR expression. The strength of this approach is that it allows changes in neuronal expression to be determined (unlike Western blotting and RT-PCR methods, which require extracts of entire ganglia and so will include glia and other non-neuronal cells). Moreover, this approach has allowed us to selectively analyse retrogradely labelled and TH-positive neurons. We acknowledge that there is unlikely to be a linear relationship between fluorescence intensity measurements and protein levels, nevertheless expression changes and the direction of those changes can be clearly identified following injury.

In contrast to GFRα2, which was selectively expressed by parasympathetic neurons, GFRα1 was expressed similarly in sympathetic and parasympathetic neurons. Our results indicate that axotomy had no effect on GFRα1 expression by pelvic parasympathetic neurons, although subtle changes may have occurred that were not measurable with our technique. However GFRα1 was down-regulated in sympathetic (TH-positive) neurons, even though these neurons do not project to the penis and therefore are unlikely to be injured. Recent studies on regeneration of injured penile axons show that GAP-43 expression is increased in some TH-positive neurons on the contralateral (uninjured) side [48], suggesting a compensatory response. This may be related to their decrease in GFRα1 expression.

## Conclusion

These studies show that there are profound changes within the pelvic ganglion after penile nerve injury. Sprouting of injured postganglionic axons occurs concurrently with structural or chemical changes in preganglionic terminals. New growth of postganglionic axon collaterals within the ganglion raises the possibility of the formation of aberrant synaptic connections between injured and un-injured ganglion neurons. Together these changes demonstrate a broader effect on the pelvic autonomic circuitry than simply loss of neuroeffector connections. These structural changes are accompanied by potential changes in neurotrophic factor signalling due to altered expression of receptors for members of the GDNF family. Together our results advance understanding of the responses of pelvic autonomic nerve circuits to injury and may assist in designing strategies for promoting regeneration.

## Methods

### Surgical procedures and tissue removal

All experiments were performed in accordance with the Code of Practice for the Care and Use of Animals for Experimental Purposes (National Health and Medical Research Council of Australia) and approved by the Animal Ethics Committees of the University of New South Wales and University of Sydney. Every attempt was made to minimise trauma and distress in the animals. A total of 16 adult male rats (6–14 weeks; outbred Wistar) were used.

All procedures were carried out under anaesthesia (48 mg/kg sodium pentobarbitone i.p., or 60 mg/kg ketamine and 10 mg/kg xylazine i.p.). Penis-projecting pelvic ganglion neurons were identified by injection of 2–5 μl of the retrograde tracer, FluoroGold (FG; 4% in distilled water; Fluorochrome, Englewood, CO, USA), into the cavernous space of the penis using a glass micropipette attached by flexible tubing to a glass syringe filled with silicon oil or an insulin syringe fitted with a 30 G needle [32,62]. Animals were allowed to recover for 7 days before further surgery; this is sufficient time for the dye to be transported to somata in the pelvic ganglia [32]. Unilateral penile nerve injury (axotomy) was then performed on 14 rats (10 penile nerve cut, 4 penile nerve crush). The pelvic ganglion on the left side was identified and the penile nerve completely transected or crushed, 1–2 mm from the body of the ganglion. Crush injury was performed at two adjacent sites, using fine forceps and holding them in place for ~3 seconds per crush; this was sufficient to cause the nerve to change its appearance at the crush site (from white to grey), indicating a successful lesion. The right ganglion was left intact. The majority of penis-projecting axons travel via the ipsilateral penile nerve [31,63], therefore most of the FG-labelled neurons in the ipsilateral pelvic ganglion would be axotomised. FG has been used extensively by many groups, and there is no evidence of a detrimental effect on neuronal health. Moreover, in the current study, all measurements of FG neurons obtained from injured ganglia were compared with FG neurons from the uninjured side of the same animal, therefore any effects of the lesion could be distinguished from any putative effects of the FG. Two rats underwent abdominal incision and visualisation of the penile nerve but no lesion was performed. Animals were allowed to recover for 7–8 days, then re-anaesthetised and perfused intra-cardially with saline containing 5000 IU/ml heparin and 1% sodium nitrite, followed by freshly made 4% paraformaldehyde in 0.1 M phosphate buffer, pH 7.4. Both pelvic ganglia were removed and pinned flat to a silicone-lined dish during overnight post-fixation, then washed in 0.1 M phosphate-buffered saline, pH 7.2 (PBS) and stored in PBS at 4°C. Ganglia were cryoprotected overnight in PBS containing 30% sucrose before cryosectioning.

### Immunohistochemistry

To reduce variability due to different immunostaining sessions, the injured and contralateral ganglia from the same animal were placed in the same block. Cryosections (14 μm) were thaw-mounted on 1% gelatin-coated slides before being processed for immunohistochemistry. To avoid double counting of somata, sections were allocated sequentially within groups of 4–6 slides so that adjacent sections were not placed on the same slide. Sections were treated with PBS containing 10% horse serum and 0.1% Triton X-100, then incubated with combinations of primary antisera (Table 1a) for 18–24 hours at room temperature in a dark, humid chamber. After washing for 15 mins in PBS, secondary antisera (Table 1b) were applied for 2–4 hours under the same conditions. For triple labelling, AMCA-avidin D (Vectorlabs, Burlingame, CA, U.S.A., 1:100) was used after exposure to biotin-labelled secondary antiserum. Sections were mounted and cover-slipped using carbonate-buffered glycerol (pH 8.6).

Antisera against the following substances were used in different combinations: GFRα1and GFRα2, the preferred receptors for GDNF and neurturin, respectively; VIP (vasoactive intestinal peptide), a marker for many parasympathetic ganglion neurons [29] and all pelvic parasympathetic neurons innervating the penis [30,32]; and TH (tyrosine hydroxylase), a marker of noradrenergic sympathetic pelvic ganglion neurons [29]. Sections of rat ganglia were triple stained for GFRα1/VIP/TH or GFRα2/VIP/TH to enable direct comparison of GFR expression in VIP and TH neurons within the same section. Antibodies against synaptic proteins (synapsin and synaptophysin) were used as markers of preganglionic terminals or potential synapses formed by newly growing fibres [36,37]. Immunostaining for TH and NOS has been described extensively in the pelvic ganglia and the expression patterns we observed closely matched previously published studies. Absorption tests were unable to be performed for the GFRα1 and GFRα2 antisera due to unavailability of the ligand to which the antibody was raised, however the GFRα2 antibody shows no immunostaining in peripheral tissues of GFRα2 knockout mice [24]. GFRα1 immunostaining does not differ between pelvic ganglia of wild-type and GFRα2 knockout mice (data not shown).

### Analysis

Sections were viewed using an Olympus BX51 microscope. Monochrome, 8-bit images of sectioned ganglia were captured for documentation and analysis using a Spot-RT camera (Diagnostic Instruments, Sterling Heights, MI, USA) and digitised using Image Pro Plus software (Version 4.5; Media Cybernetics, Carlsbad, CA, USA). Figures were produced directly from digital images. If necessary the saturation and contrast was adjusted to best resemble the native immunostaining signal.

To measure the sprouting response of parasympathetic neurons in injured ganglia, we quantified the close associations formed between VIP axons and pelvic ganglion neurons. These associations were referred to as "VIP baskets" [36,64] and were defined as where one or more VIP-positive varicose fibres closely encircled more than 50% of the soma profile of a particular neuron. Neurons within the pelvic ganglion are quite closely packed and the distance of this close association was generally hard to measure under the light microscope; it was estimated to be typically 1–3 μm. To avoid double counting, we only assessed neurons sectioned through the nucleus. We quantified VIP baskets associated with all FG-labelled (i.e. penis-projecting) neurons in lesioned and contralateral control ganglia to identify lesioned neurons targeted by sprouts after axotomy.

For quantification of GFRα1 and GFRα2 expression in axotomised somata, intensity of the immunostaining was analysed in all FG-labelled neurons that were sectioned through the nucleus. GFR expression was also assessed in tyrosine hydroxylase (TH) -positive neurons, very few or none of which project to the penis [32]. Axotomised and matching contralateral (control) ganglia were sectioned and stained at the same time. In each neuron a circular area of cytoplasm (6 μm diameter) was selected. GFR immunostaining was typically granular rather than smooth and distributed throughout the cytoplasm. Each sample was taken from a random point midway between the nucleus and the plasma membrane. A digital image was acquired using an RT Spot camera (Diagnostic Instruments, Sterling Heights, MI, USA) and the fluorescence intensity quantified using Image Pro Plus software (Version 4.5; Media Cybernetics, Carlsbad, CA, USA). Analyses of lesioned ganglia and their matched controls were performed on the same day and digital images acquired with the same exposure time, chosen to avoid saturated pixels. Because of the heterogeneity in staining intensity between neurons, it was not possible to determine which cells were completely negative, so no background subtraction was performed. Therefore all fluorescence measurements are raw measurements of intensity. Between 50 and 200 neurons were analysed per ganglion for both GFRα1 and GFRα2 immunofluorescence intensity. All FG neurons were assessed and TH neurons analysed from the same sections. There was no selection of neurons based on immunostaining intensity. Comparison of GFR staining intensity between left and right ganglia was made using paired Students *t*-test. Values are expressed as mean ± SEM, and were considered significantly different when *P *< 0.05.

## Abbreviations

FG, FluoroGold; GDNF, glial cell line-derived neurotrophic factor; GFL, glial cell line-derived neurotrophic family of ligands; NTN, neurturin; PBS, phosphate-buffered saline; TH, tyrosine hydroxylase; VIP, vasoactive intestinal peptide.

## Authors' contributions

JRK conceived the study, trained CAP in the required techniques, supervised the experiments and advised on analyses. CAP performed the majority of the experimental work. Both authors contributed to writing this manuscript.

## Figures and Tables

**Table 1:** Primary and secondary antisera.

**a. Primary Antisera**				
Antigen	Host		Source	Working Dilution

GFRα1	Goat		R&D Systems (Minneapolis, MN, USA)	1:200
GFRα2	Goat		R&D Systems	1:200
Synapsin	Rabbit		Biogenesis (Poole, UK)	1:1000
Synaptophysin	Mouse		Dako (Botany, NSW, Australia)	1:200
Tyrosine hydroxylase	Mouse		DiaSorin (Stillwater, MN, USA)	1:2000
Vasoactive intestinal peptide	Rabbit		Dia Sorin	1:2000

**b. Secondary Antisera**				

Antigen	Host	Conjugate	Source	Working Dilution

Goat IgG	Donkey	Cy3	Jackson (West Grove, PA, USA)	1:1000
Mouse IgG	Horse	Biotin	Vectorlabs (Burlingame, CA, USA)	1:200
Rabbit IgG	Donkey	FITC	Jackson	1:50
